# Supplementary material for: Identification of Biomarkers Related to Metabolically Unhealthy Obesity in Korean Obese Adolescents: A Cross-Sectional Study
Source: Children (Basel). 2023 Feb 8;10(2):322. doi: 10.3390/children10020322 (PMC9955165; doi:10.3390/children10020322)
Supplement: Supplementary file 1 [file children-10-00322-s001.zip › children-2152913-supplementary.pdf]

Table S1. The metabolites of MUO and MHO in adolescents

		Total participants (*n*=148)		
		MHO (*n*=74)	MUO (*n*=74)	P
**Acylcarnitines**[𝒻]				
Carnitine	C0	50.08 ±1.081	52.81 ±1.013	0.2932
Propionlycarnitine	C3	0.444 ±0.016	0.480 ±0.017	0.4150
Propenoylcarnitine	C3:1	0.011 ±0.000	0.010 ±0.000	0.2981
Butyrylcarnitine	C4	0.227 ±0.024	0.242 ±0.013	0.1821
Butenylcarnitine	C4:1	0.021 ±0.000	0.019 ±0.001	0.1049
Hydroxybutyrylcarnitine	C4-OH(C3-DC)	0.038 ±0.001	0.039 ±0.001	0.4453
Valerylcarnitine	C5	0.149 ±0.006	0.164 ±0.006	0.2769
Tiglylcarnitine	C5:1	0.025 ±0.001	0.026 ±0.001	0.3429
Glutaconylcarnitine	C5-DC(C6-OH)	0.018 ±0.001	0.018 ±0.001	0.9714
Glutarylcarnitine (Hydoxyhexanoylcarnitine)	C5-OH (C3-DC-M)	0.038 ±0.001	0.039 ±0.001	0.9268
Hexanoylcarnitine	C6(C4:1-DC)	0.094 ±0.003	0.102 ±0.003	0.0833
Hexenoylcarnitine	C6:1	0.018 ±0.001	0.017 ±0.000	0.1558
Pimelylcarnitine	C7-DC	0.045 ±0.002	0.046 ±0.002	0.9898
Octanoylcarnitine	C8	0.203 ±0.011	0.207 ±0.010	0.9062
Nonaylcarnitine	C9	0.030 ±0.001	0.030 ±0.001	0.4639
Decanoylcarnitine	C10	0.305 ±0.013	0.328 ±0.015	0.3715
Decenoylcarnitine	C10:1	0.243 ±0.010	0.258 ±0.010	0.5765
Decadienylcarnitine	C10:2	0.037 ±0.001	0.038 ±0.001	0.1400
Dodecanoylcarnitine	C12	0.111 ±0.005	0.118 ±0.005	0.4434
Dodecenoylcarnitine	C12:1	0.177 ±0.008	0.190 ±0.007	0.4982
Dodecanedioylcarnitine	C12-DC	0.051 ±0.001	0.051 ±0.001	0.3910
Tetradecanoylcarnitine	C14	0.045 ±0.002	0.047 ±0.001	0.3982
Tetradecenoylcarnitine	C14:1	0.143 ±0.007	0.142 ±0.005	0.9237
Hydroxytetradecenoylcarnitine	C14:1-OH	0.014 ±0.001	0.016 ±0.001	0.1369
Tetradecadienylcarnitine	C14:2	0.054 ±0.003	0.055 ±0.003	0.7395
Hydroxytetradecadienylcarnitine	C14:2-OH	0.011 ±0.000	0.011 ±0.000	0.1921
Hexadecanoylcarnitine	C16	0.148 ±0.005	0.156 ±0.004	0.3564
Hexadecenoylcarnitine	C16:1	0.051 ±0.002	0.051 ±0.001	0.8147
Hexadecadienylcarnitine	C16:2	0.020 ±0.001	0.018 ±0.001	0.7218
Hydroxyhexadecadienylcarnitine	C16:2-OH	0.012 ±0.000	0.013 ±0.000	0.2460
Octadecanoylcarnitine	C18	0.039 ±0.001	0.041 ±0.001	0.8269
Ocradecenoylcarnitine	C18:1	0.195 ±0.006	0.194 ±0.004	0.8007
Hydroxyoctadecenoylcarnitine	C18:1-OH	0.011 ±0.000	0.010 ±0.000	0.0726
Octadecadienylcarnitine	C18:2	0.123 ±0.004	0.120 ±0.004	0.1879
**Amino Acids**[𝒻]				
Arginine		95.39 ±3.928	86.03 ±3.291	0.0575
Asparagine		39.48 ±0.787	39.12 ±0.859	0.3998
Citrulline		25.52 ±0.527	26.96 ±0.656	0.2781
Glutamate		108.3 ±4.889	120.00 ±4.877	0.1244
Isoleucine		88.61 ±2.772	89.09 ±2.012	0.5758
Leucine		163.6 ±4.416	168.8 ±3.818	0.8891
Methionine		25.78 ±0.622	26.11 ±0.546	0.8443
Ornithine		100.20 ±3.130	96.85 ±2.821	0.1730
Phenylalanine		74.75 ±1.331	73.26 ±1.268	0.1795

	Total participants (*n*=148)		
	**MHO (*n*=74)**	**MUO (*n*=74)**	**P**
**Amino Acids**[ƒ]			
Proline	186.8 ±4.619	178.6 ±4.594	0.1280
Threonine	129.3 ±2.912	125.7 ±2.495	0.2238
Tryptophan	68.30 ±1.451	67.16 ±1.239	0.4684
Tyrosine	87.21 ±2.097	84.80 ±1.528	0.1634
Valine	247.2 ±5.005	248.8 ±4.672	0.7186
**Biogenic Amines**[ƒ]			
Acetylornithine	1.192 ±0.095	1.327 ±0.090	0.6188
Alpha-AAA	1.352 ±0.079	1.483 ±0.064	0.3730
Creatinine	54.28 ±1.617	54.07 ±1.405	0.3053
Petrescine	0.105 ±0.004	0.105 ±0.004	0.5659
Spermine	0.233 ±0.016	0.253 ±0.011	0.7870
Taurine	57.14 ±1.820	57.53 ±1.917	0.6572
**Glycerophospholipids**[ƒ]			
PC aa C24:0	0.189 ±0.005	0.191 ±0.004	0.1045
PC aa C26:0	0.208 ±0.011	0.231 ±0.010	0.5469
PC aa C28:1	0.290 ±0.009	0.303 ±0.010	0.4208
PC aa C30:0	3.074 ±0.119	3.047 ±0.101	0.8822
PC aa C32:0	12.83 ±0.331	13.39 ±0.328	0.2076
PC aa C32:1	16.34 ±0.738	18.48 ±0.832	0.0586
PC aa C32:3	0.414 ±0.010	0.409 ±0.011	0.7337
PC aa C34:2	281.8 ±7.496	304.1 ±7.435	0.1568
PC aa C34:3	13.02 ±0.353	14.03 ±0.418	0.1015
PC aa C34:4	1.412 ±0.049	1.454 ±0.050	0.4241
PC aa C36:2	177.7 ±3.894	190.7 ±5.233	0.2941
PC aa C36:3	99.24 ±2.784	105.70 ±2.853	0.3686
PC aa C36:4	134.9 ±3.991	143.1 ±4.110	0.4946
PC aa C36:5	19.90 ±1.045	20.08 ±0.919	0.5520
PC aa C36:6	0.846 ±0.036	0.802 ±0.025	0.6812
PC aa C38:0	3.340 ±0.102	3.198 ±0.085	0.3210
PC aa C38:3	50.25 ±1.569	55.28 ±1.659	0.0862
PC aa C38:4	88.11 ±2.800	95.94 ±3.143	0.2267
PC aa C38:5	39.70 ±1.432	42.96 ±1.393	0.2684
PC aa C38:6	91.82 ±3.034	88.25 ±2.075	0.3724
PC aa C40:1	0.400 ±0.008	0.381 ±0.008	0.1627
PC aa C40:2	0.270 ±0.006	0.276 ±0.007	0.5374
PC aa C40:3	0.555 ±0.013	0.560 ±0.015	0.4432
PC aa C40:4	3.752 ±0.147	4.217 ±0.176	0.1931
PC aa C40:5	11.71 ±0.470	13.05 ±0.493	0.0884
PC aa C40:6	37.57 ±1.498	38.34 ±1.128	0.8253
PC aa C42:0	0.557 ±0.019	0.544 ±0.017	0.2779
PC aa C42:1	0.340 ±0.010	0.317 ±0.008	0.1431
PC aa C42:2	0.276 ±0.010	0.250 ±0.007	0.0608

	Total participants (n=148)		
	MHO (n=74)	MUO (n=74)	P
**Glycerophospholipids**[f]			
PC aa C42:4	0.177 ±0.004	0.188 ±0.005	0.1406
PC aa C42:5	0.368 ±0.011	0.386 ±0.013	0.4418
PC aa C42:6	0.537 ±0.024	0.577 ±0.022	0.9490
PC ae C30:0	0.292 ±0.009	0.273 ±0.006	0.3140
PC ae C30:2	0.077 ±0.003	0.068 ±0.003	0.1721
PC ae C32:1	2.625 ±0.070	2.708 ±0.064	0.2178
PC ae C32:2	0.621 ±0.020	0.600 ±0.015	0.8911
PC ae C34:0	0.977 ±0.033	0.953 ±0.026	0.4698
PC ae C34:1	6.863 ±0.142	7.075 ±0.155	0.2898
PC ae C34:2	9.409 ±0.260	9.374 ±0.320	0.6133
PC ae C34:3	7.020 ±0.196	6.979 ±0.207	0.6551
PC ae C36:0	0.804 ±0.028	0.775 ±0.026	0.6929
PC ae C36:1	5.079 ±0.116	5.206 ±0.129	0.2934
PC ae C36:2	8.383 ±0.192	8.237 ±0.231	0.5146
PC ae C36:3	6.474 ±0.194	6.578 ±0.207	0.8941
PC ae C36:4	16.80 ±0.560	17.85 ±0.669	0.8134
PC ae C36:5	11.26 ±0.315	11.95 ±0.371	0.3616
PC ae C38:0	1.867 ±0.058	1.824 ±0.050	0.7254
PC ae C38:3	3.164 ±0.073	3.257 ±0.095	0.6020
PC ae C38:4	9.886 ±0.256	10.265 ±0.269	0.3928
PC ae C38:5	14.98 ±0.424	16.28 ±0.548	0.3578
PC ae C38:6	8.239 ±0.256	7.977 ±0.231	0.3916
PC ae C40:1	0.954 ±0.034	0.917 ±0.028	0.1659
PC ae C40:2	1.257 ±0.032	1.276 ±0.032	0.8564
PC ae C40:4	2.082 ±0.044	2.081 ±0.049	0.7202
PC ae C40:5	2.893 ±0.077	3.124 ±0.097	0.2430
PC ae C40:6	4.482 ±0.121	4.294 ±0.096	0.2473
PC ae C42:0	0.428 ±0.013	0.445 ±0.012	0.5139
PC ae C42:1	0.304 ±0.008	0.299 ±0.008	0.4641
PC ae C42:2	0.502 ±0.012	0.517 ±0.012	0.5642
PC ae C42:3	0.679 ±0.018	0.664 ±0.019	0.0996
PC ae C42:4	0.854 ±0.021	0.864 ±0.026	0.7593
PC ae C42:5	1.927 ±0.043	2.014 ±0.054	0.3711
PC ae C44:4	0.308 ±0.012	0.320 ±0.013	0.3340
PC ae C44:5	1.722 ±0.049	1.801 ±0.058	0.6401
PC ae C44:6	1.398 ±0.039	1.406 ±0.044	0.4171
lysoPC a C14:0	3.759 ±0.115	3.725 ±0.099	0.0570
lysoPC a C16:0	111.5 ±2.961	117.9 ±2.823	0.2715
lysoPC a C16:1	3.215 ±0.104	3.318 ±0.092	0.4642
lysoPC a C17:0	1.269 ±0.036	1.218 ±0.041	0.2376
lysoPC a C18:0	31.07 ±0.903	32.50 ±0.944	0.4854
lysoPC a C18:1	14.34 ±0.406	15.30 ±0.424	0.2462
lysoPC a C18:2	22.66 ±0.780	23.11 ±0.836	0.1025
lysoPC a C20:3	1.877 ±0.065	2.085 ±0.065	0.1025
lysoPC a C20:4	5.018 ±0.169	5.325 ±0.170	0.4608

	Total participants (*n*=148)		
	**MHO (*n*=74)**	**MUO (*n*=74)**	**P**
**Glycerophospholipids**[∮]			
lysoPC a C24:0	0.189 ±0.005	0.191 ±0.004	0.8313
lysoPC a C26:0	0.208 ±0.011	0.231 ±0.010	0.6207
lysoPC a C26:1	1.171 ±0.135	1.573 ±0.139	0.7292
lysoPC a C28:0	0.259 ±0.009	0.281 ±0.010	0.7855
lysoPC a C28:1	0.290 ±0.009	0.303 ±0.010	0.7282
**Sphingolipids**[∮]			
SM (OH) C14:1	4.075 ±0.088	3.860 ±0.098	0.1106
SM C16:0	101.7 ±2.178	102.7 ±2.124	0.5217
SM C16:1	2.605 ±0.060	2.495 ±0.069	0.1876
SM (OH) C16:1	18.51 ±0.291	18.28 ±0.357	0.7710
SM C18:0	23.89 ±0.708	24.51 ±0.632	0.4852
SM C18:1	12.59 ±0.305	12.34 ±0.298	0.2856
SM (OH) C22:1	10.50 ±0.333	10.88 ±0.306	0.9607
SM (OH) C22:2	8.419 ±0.184	8.141 ±0.159	0.1882

Mean ± SE. [∮]tested in the following logarithmic transformation, *P*-values were derived from independent t-test for each of the variables of the adolescents in the metabolically unhealthy obesity (MUO) group and of the adolescents in the metabolically healthy obesity (MHO) group. PC, phosphatidylcholine; lysoPC, lysophosphatidylcholine; a, acyl; aa, diacyl; ae, acyl-alkyl; SM, sphingomyelin; SM(OH), hydroxysphingomyelin

Table S2. Predictors of the MUO adolescents' prevalence ORs on significantly different metabolites

Variables	Total subjects (*n*=148) ORs (95 % CI) For MUO adolescents	P
**Quartile of acylcarnitines by the length**		
Free carnitine	1.300 (0.970–1.743)	0.0768
Short-chain acylcarnitine	1.602 (1.181–2.172)	**0.0018**
Middle-chain acylcarnitine	1.164 (0.871–1.556)	0.3029
Long-chain acylcarnitine	1.114 (0.835–1.488)	0.4620
**Quartile of sphingolipids**		
Sphingolipids	1.044 (0.783–1.393)	0.7687
**Quartile of glycerophospholipids by the type**		
Lysophosphatidylcholine acyl	1.164 (0.871–1.556)	0.3029
Phosphatidylcholine diacyl	1.300 (0.970–1.743)	0.0768
Phosphatidylcholine acyl-alkyl	1.067 (0.800–1.424)	0.6591

P-values derived from a logistic regression analysis on the metabolically unhealthy obesity (MUO). PC, phosphatidylcholine; lysoPC, lysophosphatidylcholine; a, acyl; aa, diacyl; ae, acyl-alkyl; SM, sphingomyelin; SM(OH), hydroxysphingomyelin.
